# Outcomes of ventilatory asynchrony in patients with inspiratory effort

**DOI:** 10.5935/0103-507X.20200045

**Published:** 2020

**Authors:** Frank Daniel Martos-Benítez, Yairén Domínguez-Valdés, Dailé Burgos-Aragüez, Hilev Larrondo-Muguercia, Versis Orama-Requejo, Karla Ximena Lara-Ponce, Iraida González-Martínez

**Affiliations:** 1 Unidad de Cuidados Intensivos - 8B, Hospital Clínico Quirúrgico “Hermanos Ameijeiras”, Universidad de Ciencias Médicas de La Habana - La Habana, Cuba.; 2 Unidad de Cuidados Intensivos, Hospital Universitario “Dr. Miguel Enríquez”, Universidad de Ciencias Médicas de La Habana - La Habana, Cuba.

**Keywords:** Interactive ventilatory support, Physiological monitoring, Mortality, Respiration, artificial/methods, Intensive care units, Soporte ventilatorio interactivo, Monitoreo fisiológico, Mortalidad, Respiración artificial/métodos, Unidades de cuidados intensivos

## Abstract

**Objective:**

To identify the relationship of patient-ventilator asynchrony with the level of sedation and hemogasometric and clinical results.

**Methods:**

This was a prospective study of 122 patients admitted to the intensive care unit who underwent > 24 hours of invasive mechanical ventilation with inspiratory effort. In the first 7 days of ventilation, patient-ventilator asynchrony was evaluated daily for 30 minutes. Severe patient-ventilator asynchrony was defined as an asynchrony index > 10%.

**Results:**

A total of 339,652 respiratory cycles were evaluated in 504 observations. The mean asynchrony index was 37.8% (standard deviation 14.1 - 61.5%). The prevalence of severe patient-ventilator asynchrony was 46.6%. The most frequent patient-ventilator asynchronies were ineffective trigger (13.3%), autotrigger (15.3%), insufficient flow (13.5%), and delayed cycling (13.7%). Severe patient-ventilator asynchrony was related to the level of sedation (ineffective trigger: p = 0.020; insufficient flow: p = 0.016; premature cycling: p = 0.023) and the use of midazolam (p = 0.020). Severe patient-ventilator asynchrony was also associated with hemogasometric changes. The persistence of severe patient-ventilator asynchrony was an independent risk factor for failure of the spontaneous breathing test, ventilation time, ventilator-associated pneumonia, organ dysfunction, mortality in the intensive care unit, and length of stay in the intensive care unit.

**Conclusion:**

Patient-ventilator asynchrony is a frequent disorder in critically ill patients with inspiratory effort. The patient’s interaction with the ventilator should be optimized to improve hemogasometric parameters and clinical results. Further studies are required to confirm these results.

## Abstract (es)

**Objetivo:**

Identificar la relación de la asincronía paciente-ventilador con el nivel de sedación y evaluar la asociación con los resultados hemogasométricos y clínicos.

**Métodos:**

Estudio prospectivo de 122 pacientes admitidos en la unidad de cuidados intensivos con > 24 horas de ventilación mecánica invasiva y esfuerzo inspiratorio. En los primeros 7 días de ventilación, diariamente se evaluó la asincronía paciente-ventilador durante 30 minutos. La asincronía paciente-ventilador severa se definió con un índice de asincronía > 10%.

**Resultados:**

Se evaluaron 339.652 ciclos respiratorios en 504 observaciones. La media del índice de asincronía fue 37,8% (desviación estándar 14,1% - 61,5%). La prevalencia de asincronía paciente-ventilador severa fue 46,6%. Las asincronías paciente-ventilador más frecuentes fueron: *trigger* ineficaz (13,3%), auto-*trigger* (15,3%), flujo insuficiente (13,5%) y ciclado demorado (13,7%). La asincronía paciente-ventilador severa se relacionó con el nivel de sedación (*trigger* ineficaz: p = 0,020; flujo insuficiente: p = 0,016; ciclado precoz: p = 0,023) y el uso de midazolam (p = 0,020). La asincronía paciente-ventilador severa se asoció con las alteraciones hemogasométricas. La persistencia de la asincronía paciente-ventilador severa fue un factor de riesgo independiente para fracaso en la prueba de ventilación espontánea, tiempo de ventilación, neumonía asociada al ventilador, disfunción de órganos, mortalidad en la unidad de cuidados intensivos y estadía en la unidad de cuidados intensivos.

**Conclusión:**

La asincronía paciente-ventilador es un trastorno frecuente en los pacientes críticos con esfuerzo inspiratorio. La interacción del paciente con el ventilador debe optimizarse para mejorar los parámetros hemogasométricos y los resultados clínicos. Se requieren otros estudios que confirmen estos resultados.

## INTRODUCTION

The objectives of mechanical ventilation are to improve gas exchange, reduce the work of breathing and relieve patient discomfort. Patient-ventilator asynchrony (PVA), given by the disparity between the needs of the patient and the time, flow, volume, or pressure provided by the ventilator,^(1)^ can hinder the fulfillment of these objectives. Therefore, the patient’s adaptation to the ventilator is a crucial step to achieve ventilatory goals.

In patients with invasive mechanical ventilation, the type and frequency of PVA is determined by the presence or absence of inspiratory effort. Patient-ventilator asynchrony is low in patients with optimal neuromuscular blockade, which is only used in the first hours in cases of severe respiratory compromise.^(2)^ However, the remaining ventilation period involves patients making inspiratory effort, and it is at these times that PVA is observed more frequently. The problem is more complex when considering the different ventilation modes available and the use of sedation.

Patient-ventilator asynchrony requires special attention because it is associated with an increased need for sedatives, work of breathing, injury to respiratory muscles, alterations in the ventilation/perfusion ratio, intrinsic positive end-expiratory pressure, prolonged ventilation time, prolonged stay, and higher mortality and health costs.^(3)^ Ventilator-associated lung injury (VALI) is one of the main mechanisms currently linked to clinical outcomes in ventilated patients.^(4)^ Spontaneous inspiratory effort can be superimposed to mandatory ventilation producing increased transpulmonary pressure; the relationship between spontaneous and mandatory ventilation determines alveolar aeration and pulmonary tissue strain. Patient-ventilator asynchrony exposes the lungs to greater strain, alveolar overdistension, or cyclic collapse of poorly aerated regions, which induces tissue inflammation and the development of VALI.^(5,6)^

Pioneering studies in patients with invasive ventilation focused on the analysis of the specific types of PVA at the beginning of ventilation.^(7,8)^ Recently, Blanch et al. found a stronger relationship between PVA and mortality.^(9)^ The objectives of the present study were to identify the relationship of PVA with sedation level and hemogasometric and clinical outcomes in critically ventilated patients with inspiratory effort.

## METHODS

A prospective study was conducted in the intensive care unit (ICU) 8B of the *Hospital Clínico Quirúrgico “Hermanos Ameijeiras”* from July 2017 to February 2019. This is a university center with a total of 630 beds and is a reference center in Havana, Cuba. The ICU-8B has 12 beds and provides health care to approximately 350 medical and surgical patients per year. The present study was conducted according to the principles of the Declaration of Helsinki and was approved by the Scientific Council and the Ethics Committee for Scientific Research of the hospital. Informed consent was obtained from all participating patients.

**Figure 1:**
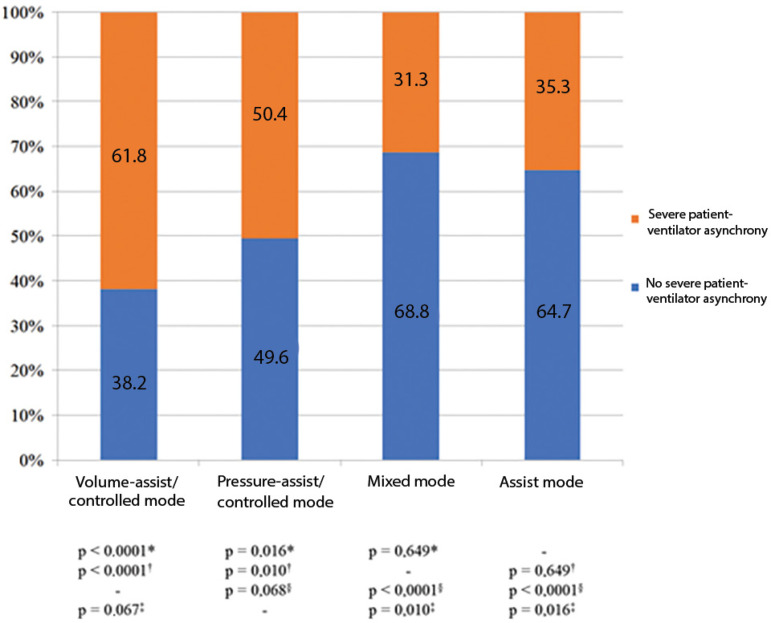
Prevalence of severe patient-ventilator asynchrony (n = 122; 504 observations). ^*^ p-value compared with the assist mode; ^†^ p-value compared with the mixed mode; ^‡^ p-value compared with the pressure-assist/controlled mode; ^§^ p-value compared with the volume-assist/controlled mode.

During the study period, 421 patients were admitted to the ICU. The 196 patients who required invasive mechanical ventilation were included. The exclusion criteria were as follows: patients with invasive ventilation ≤ 24 hours, because short ventilation periods make difficult to interpret the relationship of PVA with clinical outcomes; patients from another ICU, as health care in another ICU can affect clinical outcomes; and patients without inspiratory effort due to the use of neuromuscular blockers, neuromuscular disease, or catastrophic brain injury, as this can influence the appearance of PVA (Figure 1S - Supplementary material).

Within the first 24 hours after starting mechanical ventilation, the following variables were collected: age, sex, weight, body mass index, type of patient, reason for invasive ventilation, sepsis/septic shock, use and dose of vasoactive drugs, need for renal replacement therapy, Sequential Organ Failure Assessment (SOFA) scale, and Acute Physiology and Chronic Health Evaluation (APACHE) II scale.

Patients were ventilated with the Evita 4, Evita XL (Dräger, Lübeck, Germany), Savina (Dräger, Lübeck, Germany), Bellavista 1000 (imtmedical, Switzerland), or SERVO-air 2.1 ventilator (Maquet, Röntgenvägen, Sweden). The ICU medical team knew about the data collection but not the objectives of the research. The ventilatory adjustments and medical treatment of the patients were left to the attending physician. The presence of PVA in the first 7 days of ventilation was evaluated daily. In each evaluation, the pressure-time, flow-time, and volume-time curves were recorded digitally (Canon PowerShot SX 530 16-megapixel camera) for 30 minutes. In all cases, it was guaranteed that no diagnostic or therapeutic intervention (including modifications to the ventilatory parameters and aspiration of the artificial airway) would be performed 30 minutes before the evaluations.

In each evaluation, the following hemogasometric variables were recorded: arterial oxygen pressure (PaO_2_), arterial oxygen saturation (SaO_2_), pH, arterial pressure of carbon dioxide (PaCO_2_) and bicarbonate (HCO_3_^-^), PaO_2_/inspiratory fraction of O_2_ ratio (PaO_2_/FiO_2_), alveolar oxygen pressure (P_A_O_2_), PaO_2_/P_A_O_2_ ratio, oxygenation index (OI = FiO_2_ × mean airway pressure/PaO_2_), alveolar-to-arterial oxygen difference (D_A-a_O_2_), shunt fraction (Qs/Qt = 100 × 0.0031 × D_A-a_O_2_/(0.0031 × D_A-a_O_2_) + 5), and ventilation index (VI = RR × (peak inspiratory pressure - PEEP) × PaCO_2_/1.000). Variables related to sedation were the use and dose of sedative and the Richmond Agitation-Sedation Scale score (RASS; agitated ≥ 1 point; awake and calm/light sedation 0 to -2 points; and deep sedation ≤ -3 points).^(10)^

The clinical response variables evaluated were ΔSOFA (SOFA on the 3rd, 5th, and 7th days of ventilation - SOFA of the day of initiation of ventilation), ventilator-associated pneumonia (VAP), failure of the spontaneous breathing test (clinical or hemogasometric signs of intolerance during two hours of testing),^(11)^ ventilation time, length of ICU stay, and ICU mortality.

### Assessment of patient-ventilator asynchrony

**Figure 2:**
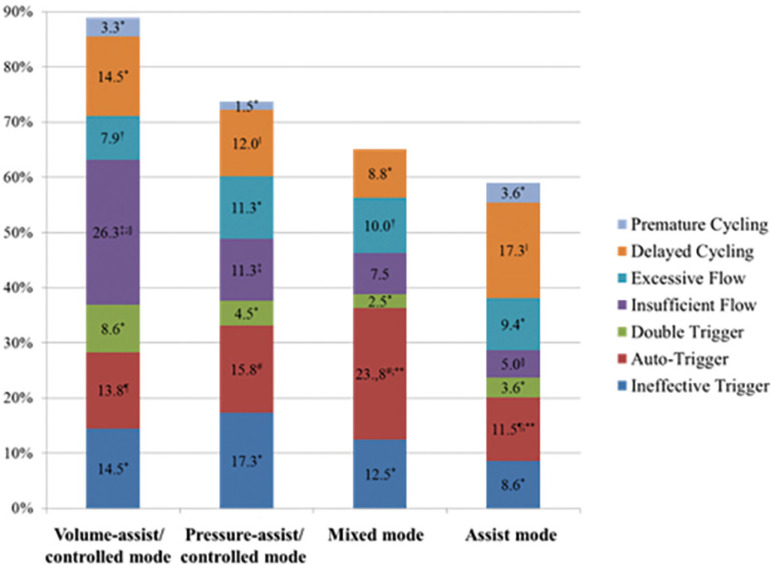
Prevalence of severe patient–ventilator asynchrony according to ventilation mode (n = 122; 504 observations). The same patient could present more than one type of severe PVA. ^*^ p > 0.05 compared with other ventilation modes; ^†^ p = 0.047 volume-assist/controlled mode versus mixed mode; ^‡^ p = 0.013 volume-assist/controlled mode versus pressure-assist/controlled; ^§^ p = 0.001 volume-assist/controlled mode versus assist mode; ^¶^ p <0.0001 volume-assist/controlled mode versus assist mode; ^||^ p = 0.009 pressure-assist/controlled mode versus assist mode; ^#^ p = 0.001 pressure-assist/controlled mode versus mixed mode; ^**^ p = 0.001 mixed mode versus assist mode.

The pressure-time, flow-time and volume-time curves were evaluated breath by breath by two different physicians to identify the presence of PVA (kappa index = 0.87). Three types of PVA and their respective subtypes were explored: trigger asynchrony (ineffective trigger, auto-trigger, and double trigger); flow asynchrony (insufficient flow and excessive flow); and cycling asynchrony (premature cycling and delayed cycling) (Figures 2S to 8S - Supplementary material).^(12,13)^ The asynchrony index (AI) was defined as the number of asynchronous events divided by the number of respiratory cycles (initiated by the patient or by the ventilator) and multiplied by 100; AI ≥ 10% was used to identify patients with severe PVA. This value was associated with poor results in previous studies.^(14)^

The duration of severe PVA can influence clinical outcomes, so persistent severe PVA was defined as AI ≥ 10% on the day of ventilation that persisted on days 3, 5, and 7 of ventilation.

### Statistical analysis

For the statistical analysis, the ventilation modes were grouped into volume- assist/controlled mode (V-A/C): volume-controlled ventilation + trigger (Bellavista 1000 and SERVO-air 2.1) and intermittent positive pressure ventilation (IPPV) + trigger (Evita 4, Evita XL and Savina); pressure-assist/controlled mode (P-A/C): pressure-controlled ventilation + trigger (Bellavista 1000 and SERVO-air 2.1), pressure-regulated volume-controlled ventilation + trigger (SERVO-air 2.1), and IPPV with autoflow + trigger (Evita 4, Evita XL and Savina); mixed mode: ventilation with bilevel positive airway pressure + pressure-supported ventilation (PSV) (Evita 4, Evita XL and Savina, Bellavista 1000 and SERVO-air 2.1) and synchronized intermittent mandatory ventilation + PSV (Evita 4, Evita XL and Savina, Bellavista 1000 and SERVO-air 2.1); and 4); and assist mode: PSV (Evita 4, Evita XL and Savina, Bellavista 1000 and SERVO-air 2.1).

The categorical variables are shown as counts and percentages. The quantitative variables are expressed as the mean with standard deviation (SD) or median with interquartile range (IQR), according to the normality of the population (evaluated with the Kolmogorov-Smirnov test and the Q-Q graph). Differences between groups were evaluated using the chi-squared (χ^2^) test with Yates correction and Student’s *t-*test for qualitative and quantitative variables, respectively.

To evaluate the relationship between sedation and severe PVA, a sensitivity analysis was performed to examine the individual and combined effects of sedative drugs. A subgroup analysis was also performed to explore the influence of the level of sedation on the AI of each PVA subtype, for which a one-way analysis of variance was performed. The homoscedasticity between the groups was verified with the Levene´s test. *Post hoc* Bonferroni correction was done to evaluate the differences in the mean AI between the particular categories of sedation level.

A multivariate logistic regression (MLR) model was used to identify the factors associated with mortality in the ICU. Variables with a p-value ≤ 0.05 in the univariate analysis were included in the initial model. The automated variable selection method by backward elimination was used. The results are shown as odds ratio (OR) with respective 95% confidence interval (95%CI) and p-value.

The impact of persistent severe PVA on clinical outcomes was evaluated by a multiple linear regression model for quantitative response variables with a normal distribution and by an MLR model for binary response variables. In both models, the variables associated with mortality in the ICU were used as confounding variables. The results of the MLR model are shown as described, and the multiple linear regression model uses the regression coefficient β, 95%CI, and p-value.

Statistical hypothesis tests were considered significant with a bilateral p-value < 0.05. The statistical analysis was performed with the IBM^®^ SPSS^®^ 23.0 program (IBM, Armonk, NY, USA).

## RESULTS

A total of 122 patients with a mean age of 62.0 years (SD 15.9 years) were analyzed. Fifty-nine percent were admitted to the ICU for nonsurgical causes. A total of 60.7% of patients (n = 74) had sepsis; of these, 68.9% (n = 51) had septic shock and were given a mean daily dose of norepinephrine of 0.32µg/kg/minute (SD 0.24µg/kg/minute). A total of 19.7% of patients had pneumonia, and 36.9% had acute respiratory distress syndrome. Renal replacement therapy was required in 6.6% of cases. The average score on the SOFA scale was 5.5 points (SD 2.8 points) and on the APACHE II scale 19.1 points (SD 6.5 points). Table 1 shows the general characteristics of the patients. The type of patient, sepsis, APACHE II score, and SOFA score were independent risk factors for death in the ICU (Tables 1S and 2S - Supplementary material).

**Table 1:** Characteristics of patients on the day of starting invasive mechanical ventilation

Characteristic	
Age, years	62.0 ± 15.9
Male sex	82 (67.2)
Weight, kg	76.4 (11.7)
BMI, kg/m^2^	23.2 (6.3)
Type of patient	
Surgical	50 (41.0)
Nonsurgical	72 (59.0)
Reason for invasive mechanical ventilation *	
Septic shock	51 (41.8)
Pneumonia	24 (19.7)
Aspiration	4 (3.3)
Acute respiratory distress syndrome	45 (36.9)
Postoperative	26 (21.3)
Exacerbation of COPD/asthma	7 (5.7)
Cardiogenic pulmonary edema	12 (9.8)
Disorder of consciousness	21 (17.2)
SOFA, points	5.5 (2.8)
APACHE II, points	19.1 (6.5)
Renal replacement therapy	8 (6.6)
Mechanical ventilation time, days	9.5 (9.9)
CU stay, days	10.6 (9.6)
Mortality in the ICU	88 (72.1)

BMI - body mass index; COPD - chronic obstructive pulmonary disease; SOFA - *Sequential Organ Failure Assessment*; APACHE - *Acute Physiology and Chronic Health Evaluation*; ICU - intensive care unit.

* The same patient could present more than one reason for requiring invasive mechanical ventilation. The results are expressed as the mean ± standard deviation or n (%).

The mean ventilation time was 9.5 days (SD 9.9 days). A total of 504 observations were made, with a median of 4.0 observations per patient (IQR 2.0 - 5.0 observations) and a total of 339,652 respiratory cycles (2,784 per patient and 674 per observation). Seventy-eight observations (15.5%) were made in tracheostomized patients.

### Frequency of severe patient-ventilator asynchrony

Of the 504 observations, 152 (30.2%) were performed in volume-assist/controlled mode, 133 (26.4%) in pressure-assist/controlled mode, 80 (15.9%) in mixed mode, and 139 (27.6%) in assist mode.

The mean AI was 37.8% (SD 14.1-61.5%). In 235 observations (46.6%), severe PVA was found. In 73.2% of these, more than one type of PVA was detected. Severe PVA was more frequent in the volume-assist/controlled mode (61.8%) and in the pressure-assist/controlled mode (50.4%) (Figure 1). The prevalence of severe PVA subtypes was ineffective trigger 13.3%, auto-trigger 15.3%, double trigger 5.2%, insufficient flow 13.5%, excessive flow 9.5%, delayed cycling 13.7%, and premature cycling 2.4%. Figure 2 shows the prevalence of severe PVA subtypes according to ventilation mode.

### Relationship between sedation and severe patient-ventilator asynchrony

A total of 230 (45.6%) observations were made in patients with sedative infusion. The average score on the RASS scale was -3.85 points (SD 1.65 points). In the 504 observations, the use of sedatives was not associated with the frequency of severe PVA (112/235; 47.7% *versus* 118/269; 43.9%; p = 0.394). Among the 230 observations performed in sedated patients, the level of sedation (p = 0.368) and the score on the RASS scale (p = 0.607) were not associated with severe PVA (Table 2). However, when the PVA subtypes were evaluated, a relationship was observed between the level of sedation and PVA due to ineffective triggering (p = 0.020), insufficient flow (p = 0.016), or premature cycling (p = 0.023) (Table 3S - Supplementary material). In the Bonferroni *post hoc* analysis, it was found that the frequency of PVA due to ineffective trigger was significantly higher in patients with deep sedation, while PVA due to insufficient flow and premature cycling was associated with agitated patients (Figures 9S and 10S - Supplementary material).

**Table 2:** Relationship between sedo-analgesia and severe patient-ventilator asynchrony

Variables	Total N_obs_ = 230	Severe PVA N_obs_ = 112	No severe PVA N_obs_ = 118	p value
Level of sedation				0.368
Agitated	30 (13.0)	11 (9.8)	19 (16.1)	
Light sedation	99 (43.0)	50 (44.6)	49 (41.5)	
Deep sedation	101 (43.9)	51 (45.5)	50 (42.4)	
RASS scale, points	-3.85 ± 1.65	-3.8 ± 1.6	-3.9 ± 1.7	0.607
Midazolam	186 (80.9)	98 (87.5)	88 (74.6)	0.020
Dose, mg/kg/hour	0.17 ± 0.08	0.18 ± 0.08	0.16 ± 0.08	0.015
Propofol	52 (22.6)	17 (15.2)	35 (29.7)	0.014
Dose, mg/kg/hour	1.64 ± 0.52	1.82 ± 0.50	1.56 ± 0.51	0.082
Fentanyl	16 (7.0)	9 (8.0)	7 (5.9)	0.713
Dose, mg/kg/hour	6.6 ± 5.4	0.14 ± 0.09	0.07 ± 0.09	0.149
Ketamine	78 (33.9)	35 (31.3)	43 ± 36.4)	0.489
Dose, mg/kg/hour	1.07 ± 0.50	1.07 ± 0.51	1.07 ± 0.49	0.992
Sensitivity analysis				
Midazolam only	90 (39.1)	53 (47.3)	37 (31.4)	0.019
Propofol only	26 (11.3)	9 (8.0)	17 (14.4)	0.188
Fentanyl only *	0 (0.0)	0 (0.0)	0 (0.0)	-
Ketamine only	14 (6.1)	3 (2.7)	11 (9.3)	0.067
Midazolam + fentanyl	14 (6.1)	9 (8.0)	5 (4.2)	0.353
Midazolam + ketamine	60 (26.1)	30 (26.8)	30 (25.4)	0.932
Midazolam + propofol	22 (9.6)	6 (5.4)	16 (13.6)	0.059
Propofol + fentanyl *	0 (0.0)	0 (0.0)	0 (0.0)	-
Propofol + ketamine	4 (1.7)	2 (1.8)	2 (1.7)	1.000

PVA - patient-ventilator asynchrony; Nobs -number of observations; RASS scale - Richmond Sedation and Agitation Scale.

* There were no cases with fentanyl alone or propofol + fentanyl. The results are expressed as n (%) or the mean ± standard deviation.

The use of midazolam (p = 0.020) and its dose (p = 0.015) were associated with a higher frequency of severe PVA, while propofol (p = 0.014) was associated with a lower frequency. The use and dose of fentanyl or ketamine was not significantly related to severe PVA (Table 2). In the sensitivity analysis, it was found that midazolam infusion (p = 0.019) was the only drug associated with a higher frequency of severe PVA (Table 2).

### Relationship of severe patient-ventilator asynchrony with hemogasometric variables

All subtypes of severe PVA were related to hemogasometric changes (Table 3). Compared with patients without PVA, all those who developed severe asynchrony (with the exception of PVA due to excessive flow) had a significantly lower PaO_2_/FiO_2_ ratio. Patients with severe PVA due to insufficient flow and delayed cycling showed significant differences in almost all hemogasometric variables compared with patients without severe PVA (Table 3).

**Table 3:** Relationship of severe patient-ventilator asynchrony with hemogasometric variables (n = 122; 504 observations)

**Severe trigger asynchronies**							
**Variables**	**No severe PVA (N_obs_ = 269)**	**Severe PVA due to ineffective trigger**		**Severe PVA by auto-trigger**		** Severe PVA by double trigger**	
		**N_obs_ = 67**	p value*	**N_obs_ = 77**	p value*	**N_obs_ = 26**	p value*
Hemogasometric parameters							
pH	7.41 ± 0.07	7.40 ± 0.05	0.182	7.42 ± 0.09	0.370	7.43 ± 0.06	0.160
PaO_2_	137.1 ± 42.9	136.0 ± 43.3	0.851	135.2 ± 47.0	0.738	119.6 ± 41.0	0.047
SaO_2_	98.5 ± 1.7	98.4 ± 2.1	0.719	98.1 ± 2.4	0.175	96.0 ± 3.7	0.002
PaCO_2_	37.0 ± 10.1	35.3 ± 10.1	0.219	36.6 ± 10.0	0.759	35.3 ± 5.3	0.166
HCO_3_^-^	23.0 ± 7.0	21.9 ± 6.0	0.238	22.7 ± 6.9	0.740	22.7 ± 3.3	0.700
Respiratory indices							
PaO_2_/FiO_2_	307.5 ± 107.0	280.7 ± 85.6	0.032	282.5 ± 86.2	0.036	256.3 ± 100.7	0.020
PAO_2_	284.8 ± 81.7	313.2 ± 90.9	0.013	305.2 ± 82.9	0.055	293.9 ± 59.2	0.580
PaO_2_/PAO_2_	0.50 ± 0.18	0.46 ± 0.14	0.051	0.47 ± 0.11	0.073	0.42 ± 0.17	0.031
OI	4.9 ± 2.3	4.6 ± 3.3	0.484	4.3 ± 3.0	0.108	5.0 ± 3.1	0.874
D_A-a_O_2_ (obs.- exp.)	127.1 ± 93.0	133.4 ± 69.2	0.537	133.1 ± 70.2	0.542	143.3 ± 73.0	0.389
Qs/Qt	9.3 ± 3.6	9.0 ± 4.7	0.627	8.9 ± 5.1	0.521	9.6 ± 3.6	0.685
Ventilation index	13.6 ± 7.9	13.0 ± 7.9	0.578	15.1 ± 9.1	0.157	14.8 ± 5.1	0.287
**Severe flow asynchronies**							
**Variables**	**Severe PVA due to insufficient flow**			**Severe PVA due to excessive flow**			
	**N_obs_ = 68**	p value*		**N_obs_ = 48**		p value*	
Hemogasometric parameters							
pH	7.45 ± 0.08	< 0.0001		7.44 ± 0.09		0.032	
PaO_2_	144.7 ± 56.2	0.301		142.6 ± 51.1		0.428	
SaO_2_	97.6 ± 3.0	0.020		98.2 ± 1.4		0.249	
PaCO_2_	31.8 ± 7.2	< 0.0001		33.4 ± 9.9		0.023	
HCO_3_^-^	18.8 ± 4.8	< 0.0001		20.6 ± 6.7		0.028	
Respiratory indices							
PaO_2_/FiO_2_	271.5 ± 102.0	0.013		303.0 ± 103.0		0.787	
PAO_2_	316.7 ± 85.5	0.005		293.3 ± 52.7		0.352	
PaO_2_/PAO_2_	0.43 ± 0.16	0.004		0.49 ± 0.16		0.719	
OI	5.1 ± 2.9	0.599		4.8 ± 3.6		0.853	
D_A-a_O_2_ (obs. - exp.)	165.0 ± 87.7	0.003		127.4 ± 64.9		0.978	
Qs/Qt	10.9 ± 4.5	0.008		8.4 ± 3.4		0.109	
Ventilation index	17.3 ± 7.0	< 0.0001		14.4 ± 7.6		0.516	
**Severe cycling asynchronies**							
**Variables**	**Severe PVA due to delayed cycling**			**Severe PVA due to premature cycling**			
	**N_obs_ = 69**	p value*		**N_obs_ = 12**		p value*	
Hemogasometric parameters							
pH	7.38 ± 0.08	0.002		7.37 ± 0.09		0.057	
PaO_2 _	119.4 ± 41.5	0.002		124.5 ± 47.1		0.322	
SaO_2 _	95.4 ± 1.5	< 0.0001		98.0 ± 1.3		0.316	
PaCO_2_	45.6 ± 9.6	< 0.0001		41.6 ± 12.9		0.128	
HCO_3_-	24.9 ± 5.8	0.038		25.8 ± 7.7		0.178	
Respiratory indices							
PaO_2_/FiO_2_	274.9 ± 101.4	0.023		238.9 ± 64.7		0.029	
PAO_2 _	314.7 ± 94.8	0.009		302.2 ± 46.1		0.241	
PaO_2_/PAO_2_	0.45 ± 0.17	0.038		0.40 ± 0.10		0.006	
OI	6.7 ± 4.5	0.009		5.2 ± 2.9		0.662	
D_A-a_O_2_ (obs.- exp.)	152.8 ± 80.5	0.036		147.2 ± 26.9		0.047	
Qs/Qt	11.2 ± 4.7	0.002		9.9 ± 1.2		0.158	
Ventilation index	16.1 ± 7.1	0.017		15.9 ± 6.7		0.322	

* p value compared with "No severe PVA". PVA - patient-ventilator asynchrony; Nobs -number of observations; PaO_2_ - arterial oxygen pressure; SaO_2_ - arterial oxygen saturation; PaCO_2_ - arterial pressure of carbon dioxide; HCO_3_^-^ - bicarbonate; FiO_2_ - inspiratory fraction of O_2_; PAO_2_ - alveolar oxygen pressure; OI - oxygenation index; D_A-a_O_2_ (obs. - exp.) - observed - expected alveolar - arterial difference of oxygen; Qs/Qt - shunt fraction.

### Relationship of persistent severe patient-ventilator asynchrony with clinical outcomes

On the first day of ventilation, 44.3% (n = 54/122) of patients presented severe PVA. The persistence of severe PVA on the 3rd, 5th, and 7th days of ventilation was observed in 37.7% (n = 46/122), 30.3% (n = 37/122), and 22.1% (n = 27/122) of the cases, respectively. The relationship of persistent severe PVA and clinical outcomes was analyzed (Table 4 and Table 4S - Supplementary material). In the multivariate analysis, the presence of severe PVA on the first day of ventilation was associated with longer ventilation time (p = 0.032) and increased mortality in the ICU (p = 0.019) (Table 4A). The persistence of severe PVA on the 3rd, 5th, and 7th days of ventilation was associated with higher ΔSOFA, ventilation time, VAP, length of ICU stay, and ICU mortality (Table 4).

**Table 4:** Relationship of persistent severe patient-ventilator asynchrony with clinical results (multivariate analysis)

**Severe patient-ventilator asynchrony on the 1^st^ day of invasive mechanical ventilation and persisting on the 3^rd^ day**						
**Variables**	**1^st^ day (n = 122)**			**3^rd^ day (n = 92)**		
	OR/β*	**95%CI**	**p value**	OR/β*	**95%CI**	**p value**
SOFA ^†^	-	-	-	0.54	0.03 - 1.05	0.039
Ventilator-associated pneumonia ^‡^	1.96	0.75 - 5.07	0.168	2.48	0.91 - 6.79	0.076
Failed SBT ^‡^	1.56	0.54 - 4.50	0.415	3.73	0.88 - 15.79	0.073
Ventilation time ^†^	4.02	0.35 - 7.70	0.032	4.12	0.04 - 8.20	0.048
ICU stay ^†^	2.83	-0.82 - 6.47	0.127	5.92	1.85 - 10.0	0.005
Mortality in the ICU ^‡^	4.88	1.30 - 18.47	0.019	3.74	1.28 - 10.94	0.016
**Persistent severe patient-ventilator asynchrony on the 5^th^ and 7^th^ day of invasive mechanical ventilation**						
**Variables**	**5^th^ day (n = 72)**			**7^th^ day (n = 50)**		
	OR/β*	**95%CI**	**p value**	OR/β*	**95%CI**	**p value**
**Δ**SOFA ^†^	1.58	0.79 - 2.37	< 0.0001	1.50	0.57 - 2.42	0.002
Ventilator-associated pneumonia ^‡^	4.29	1.34 - 13.74	0.014	5.50	1.26 - 24.06	0.024
Failed SBT ^‡^	4.70	1.01 - 21.89	0.048	5.66	0.93 - 34.92	0.060
Ventilation time ^†^	4.81	0.13 - 9.48	0.044	6.15	0.18 - 12.11	0.044
ICU stay ^†^	4.91	0.57 - 9.24	0.027	6.65	1.24 - 12.07	0.017
Mortality in the ICU ^‡^	12.54	3.17 - 49.58	< 0.0001	6.94	1.43 - 33.70	0.016

OR - odds ratio; ***β*** - multiple linear regression coefficient; CI - confidence interval; SOFA - *Sequential Organ Failure Assessment*; SBT - spontaneous breathing test; ICU - intensive care unit.

* Odds ratio for multivariate logistic regression analysis and ***β*** regression coefficient for multiple linear regression analysis.

† Adjusted p-value for the type of patient, sepsis, *Acute Physiology and Chronic Health Evaluation* II score, and *Sequential Organ Failure Assessment* score by multiple linear regression.

‡ Adjusted p-value for the type of patient, sepsis, Acute *Physiology and Chronic Health Evaluation* II score, and *Sequential Organ Failure Assessment* score using multivariate logistic regression.

## DISCUSSION

In the present prospective study, a varied cohort of critically ill ventilated patients with inspiratory effort was analyzed. The incidence of acute respiratory distress syndrome was higher than that described by Bellani et al. in a recent multinational study (36.9% *versus* 23.4%),^(15)^ which could be due to the high frequency of cases with risk factors,^(16)^ such as septic shock, pneumonia, and aspiration. Mortality in the ICU was high, even if the initial mean values of SOFA and APACHE II are considered, which was related to the complexity of the patients analyzed (e.g., 41.8% in septic shock, 36.9% with acute respiratory distress syndrome) and the appearance of complications associated with prolonged ventilation (incidence of VAP: 32.0%). These data are in line with the recent evidence of high mortality rates in patients with septic shock, acute respiratory distress syndrome, and VAP.^(16-18)^

Many respiratory cycles were explored, and 38% of them were asynchronous. In previous studies, the frequency of PVA oscillated between 3% and 38%, depending on the asynchrony detection method, the type of PVA investigated, the ventilatory mode, the presence of inspiratory effort, and respiratory mechanisms.^(9,19-24)^

The frequency of severe PVA was higher than that described by other authors.^(7-9)^ These results are explained by the high frequency of respiratory disorders or septic shock in the patients analyzed, as well as the study design: only patients with inspiratory effort, analysis of a wide variety of types of PVA in various ventilation modes, and the long evaluation period (7 days). Most studies on PVA have analyzed patients who were relatively stable or had only one respiratory disorder in a few ventilation modalities (sometimes including cases with neuromuscular blockade) and who were observed for a short period.^(7-9,19,22,23)^ In this study, evaluations were performed for several consecutive days, which represents the real context of the clinical course (day to day) of critically ill patients; consequently, we had a greater probability of detecting PVA.

An important finding of the present study was the association between deep sedation and PVA due to ineffective triggering. Recent studies have also described a higher frequency of PVA in patients with deep sedation than those who had light sedation.^(1,25)^ Vignaux et al. showed that the ineffective trigger can be unnoticed during deep sedation.^(26)^ Therefore, clinical examination and analysis of ventilatory curves is mandatory. Moreover, because deep sedation is an independent risk factor for hospital death (OR 2.36; 95%CI 1.31 - 4.25),^(27)^ interaction with PVA may contribute to worsening clinical outcomes.

The drugs used for sedation can affect the impulse and respiratory pattern, as well as decrease the effort of the respiratory muscles during ventilation.^(28)^ Therefore, in addition to the level of sedation, the type of drug used can influence the incidence of PVA. As in other regions,^(29)^ midazolam was the most commonly used hypnotic in this study and the sedative drug that was most linked with the presence of severe PVA. Recently, de Haro et al. observed that sedatives (e.g., midazolam, propofol, lorazepam) alone or combined with opioids (e.g., morphine, fentanyl) did not improve the frequency of PVA over opioids alone. Additionally, optimization of opioid dose was associated with lower AI^(30)^ because opioids decrease neural expiratory time and respiratory rate, with little effect on inspiratory impulse or PVA.^(31)^ In a recent clinical trial in patients with difficult weaning, Conti et al. observed that light sedation with propofol or dexmedetomidine improved patient-ventilator synchrony.^(32)^ Therefore, although there is a lack of evidence to judge the individual effect of sedative drugs, the most important factor to consider is the level of sedation. It is important to note that Chanques et al. demonstrated that changes in ventilatory parameters were more effective than changes in the level of sedation in reducing the frequency of severe PVA.^(33)^ This suggests that in patients with PVA, the infusion of sedative drugs should only be indicated after optimizing the ventilatory parameters and controlling clinical problems such as pain, anxiety, delirium, or fever. For reasons of patient safety, bolus administration of sedatives is also justified when it is evident the patient is struggling with the ventilator.^(1)^

No previous study has aimed to evaluate the association between PVA and hemogasometric disorders, so knowledge about it is limited and comes from secondary analyses. Sometimes, patients with clinically significant hemogasometric changes (e.g., PaO_2_/FiO_2_ < 150mmHg) were even excluded.^(8)^ Yonis et al. found that the reduction of AI by neurally adjusted ventilatory assist, compared with PSV, was associated with an increase in PaO_2_ (from 66.7mmHg to 77.4mmHg) and in the PaO_2_/FiO_2_ ratio (from 203mmHg to 254mmHg).^(34)^ In the present study, severe PVA affected hemogasometric parameters. Particularly important is the low PaO_2_/FiO_2_ ratio observed with most types and subtypes of severe PVA. Therefore, PVA should be controlled before assessing the severity of respiratory dysfunction. This may modify the epidemiology of acute respiratory distress syndrome and SOFA score.^(35,36)^

Patient-ventilator asynchrony has been associated with poor clinical outcomes. Schmidt et al. observed that V-A/C ventilation was associated with a greater sensation of dyspnea (OR 4.77; 95%CI 1.60 - 4.3), which improved in 35% of patients after adjustment of ventilatory parameters. Additionally, the lack of improvement in dyspnea was related to failure of extubation (17% *versus* 40%; p = 0.034).^(37)^ This suggests that inadequately low inspiratory flow or tidal volume can cause dyspnea and asynchrony, which hinders the weaning process. Severe trigger asynchronies were correlated with longer ventilation time, ICU stay, and hospital stay in early studies.^(7,8)^ Blanch et al. observed that patients with an AI > 10% had a higher mortality rate in the ICU (14% *versus* 67%) and hospital (23% *versus* 67%), as well as a longer ventilation time (6 days *versus* 16 days).^(9)^ In these studies, only the presence/absence of severe PVA and its relationship with clinical outcomes were analyzed. In the present investigation, not only was the presence/absence of severe PVA associated with poor clinical outcomes (e.g., ventilation time and mortality with severe PVA on the first day of ventilation), but persistence during ventilation days was a more powerful prognostic factor (e.g., organ dysfunction, VAP, ventilation time, ICU stay, and ICU mortality).

Insufficient ventilatory support causes damage to the respiratory muscles by increasing the work of breathing and muscle fatigue, while excessive ventilatory support produces atrophy and apoptosis of muscle fibers.^(12)^ The ineffective *trigger* during expiration produces eccentric contraction of the diaphragmatic muscle fibers and damage to the respiratory muscles,^(38)^ which explains the failure of the spontaneous breathing test, the prolongation of the ventilation time,^(39)^ and the consequent VAP.^(18,40)^ Sepsis and local inflammation contribute to organ dysfunction, prolonged stay, and mortality.^(41)^

The strengths of the study were that precise definitions of PVA were used based on the criteria currently in force;^(12)^ most types and subtypes of PVA were analyzed; the study was conducted in a center with a high standard of health care and in an ICU with qualified intensivists 24 hours a day, seven days a week; the study addresses a frequent problem in the care of critically ill patients with poorly defined clinical consequences at present.

The study also has limitations to take into account. First, no automatic asynchrony detection software was used, so human error could be present in their visual detection. Second, it was a monocentric study, so it can be difficult to generalize the results to other ICU with different characteristics. Third, a mixed cohort of surgical and nonsurgical patients with several clinical and pathophysiological disorders was analyzed, which could influence the results. Fourth, only daily evaluations were made that lasted 30 minutes. The presence and magnitude of PVA between evaluations could have an impact on clinical outcomes. Fifth, the patients were sedated with benzodiazepines, which could influence the frequency of PVA. Finally, the inspiratory effort of the patients was not objectively measured, so asynchrony by reverse trigger or delayed trigger was not evaluated, which could have been present and influenced the results. The work of breathing of the patients was also not evaluated, which is valuable information for a holistic interpretation of the pathophysiological disorders associated with PVA.

## CONCLUSION

Patient-ventilator asynchrony is a frequent disorder in patients with inspiratory effort, which is influenced by the level of sedation and type of sedative drugs. The association of patient-ventilator asynchrony with hemogasometric changes and clinical outcomes suggests the need for an active and frequent surveillance for its correction. Additional studies are required to confirm these results.
