# Supplementary material for: Outcomes of ventilatory asynchrony in patients with inspiratory effort
Source: Rev Bras Ter Intensiva. 2020 Apr-Jun;32(2):284–94. doi: 10.5935/0103-507X.20200045 (PMC7405741; doi:10.5935/0103-507X.20200045)
Supplement: Supplementary file 1 [file rbti-32-02-0284-suppl01.pdf]

Outcomes of ventilatory asynchrony in patients with inspiratory effort

Resultados de la asincronía ventilatoria en pacientes con esfuerzo inspiratorio

Frank Daniel Martos-Benítez[1], Yairén Domínguez-Valdés[1], Dailé Burgos-Aragüez[1], Hilev Larrondo-Muguercia[1], Versis Orama-Requejo[1], Karla Ximena Lara-Ponce[1], Iraida González-Martínez[2]

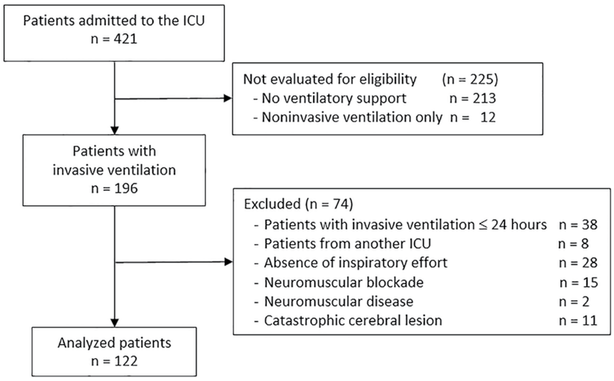

Figure 1S - Flowchart of the participants. ICU - intensive care unit.

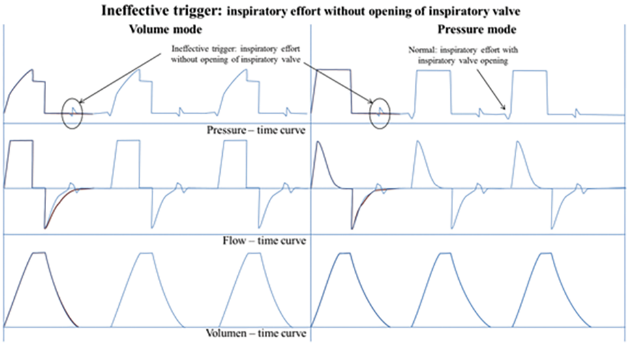

Figure 2S - Example of patient–ventilator asynchrony due to ineffective triggers. In red, the normal curves; in blue, the curves with patient–ventilator asynchrony by ineffective trigger.

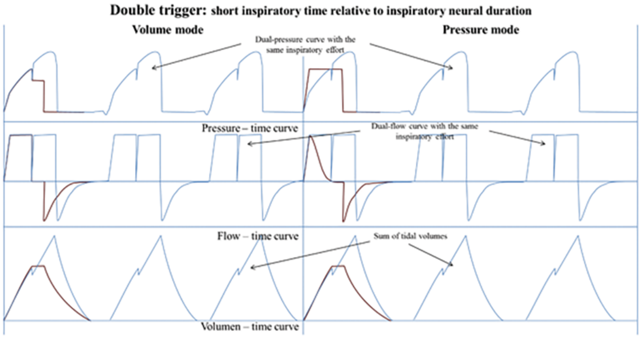

Figure 3S - Example of patient–ventilator asynchrony due to double trigger. In red, the normal curves; in blue, the curves with patient–ventilator asynchrony by double trigger.

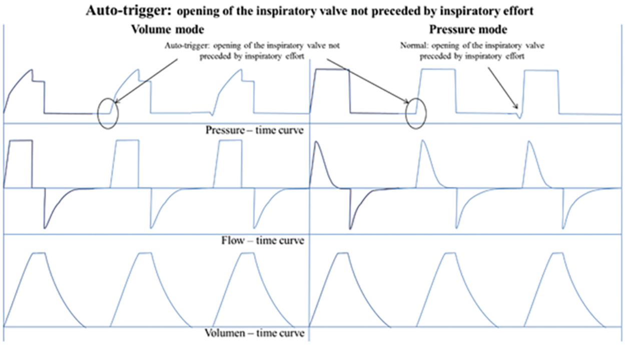

Figure 4S - Example of patient–ventilator asynchrony due to auto-trigger. In red, the normal curves; in blue, the curves with patient–ventilator asynchrony by auto-trigger.

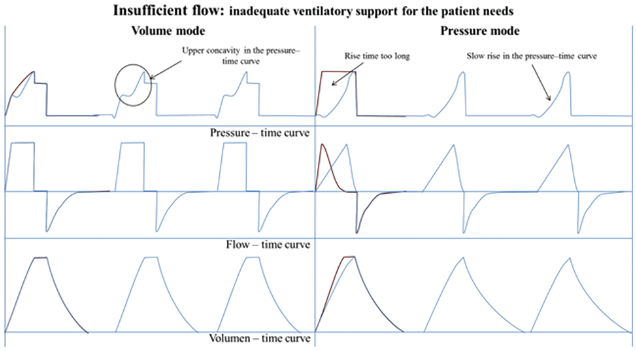

Figure 5S - Example of patient–ventilator asynchrony due to insufficient flow. In red, the normal curves; in blue, the curves with patient–ventilator asynchrony by insufficient flow.

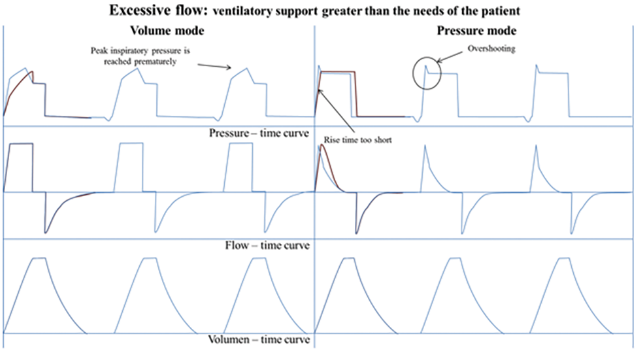

Figure 6S - Example of patient–ventilator asynchrony due to excessive flow. In red, the normal curves; in blue, the curves with patient–ventilator asynchrony by excessive flow.

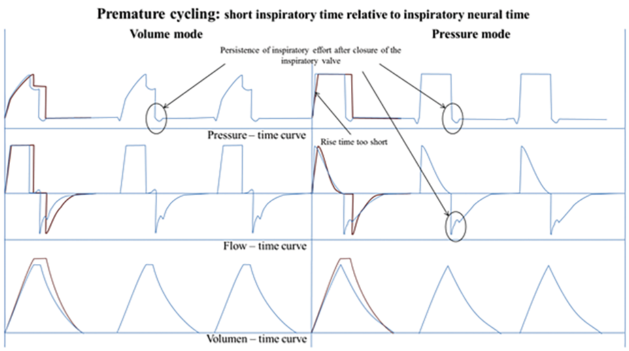

Figure 7S - Example of patient–ventilator asynchrony due to premature cycling. In red, the normal curves; in blue, the curves with patient–ventilator asynchrony by premature cycling.

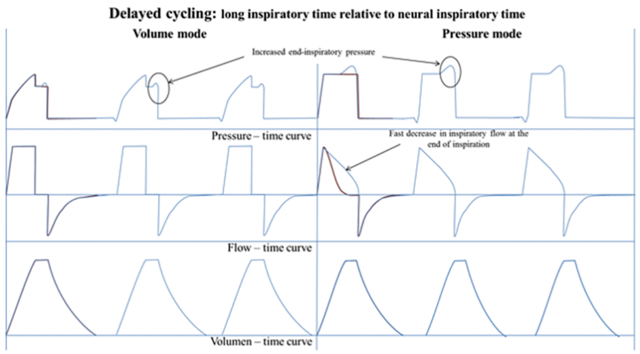

Figure 8S - Example of patient–ventilator asynchrony due to delayed cycling. In red, the normal curves; in blue, the curves with patient–ventilator asynchrony by delayed cycling.

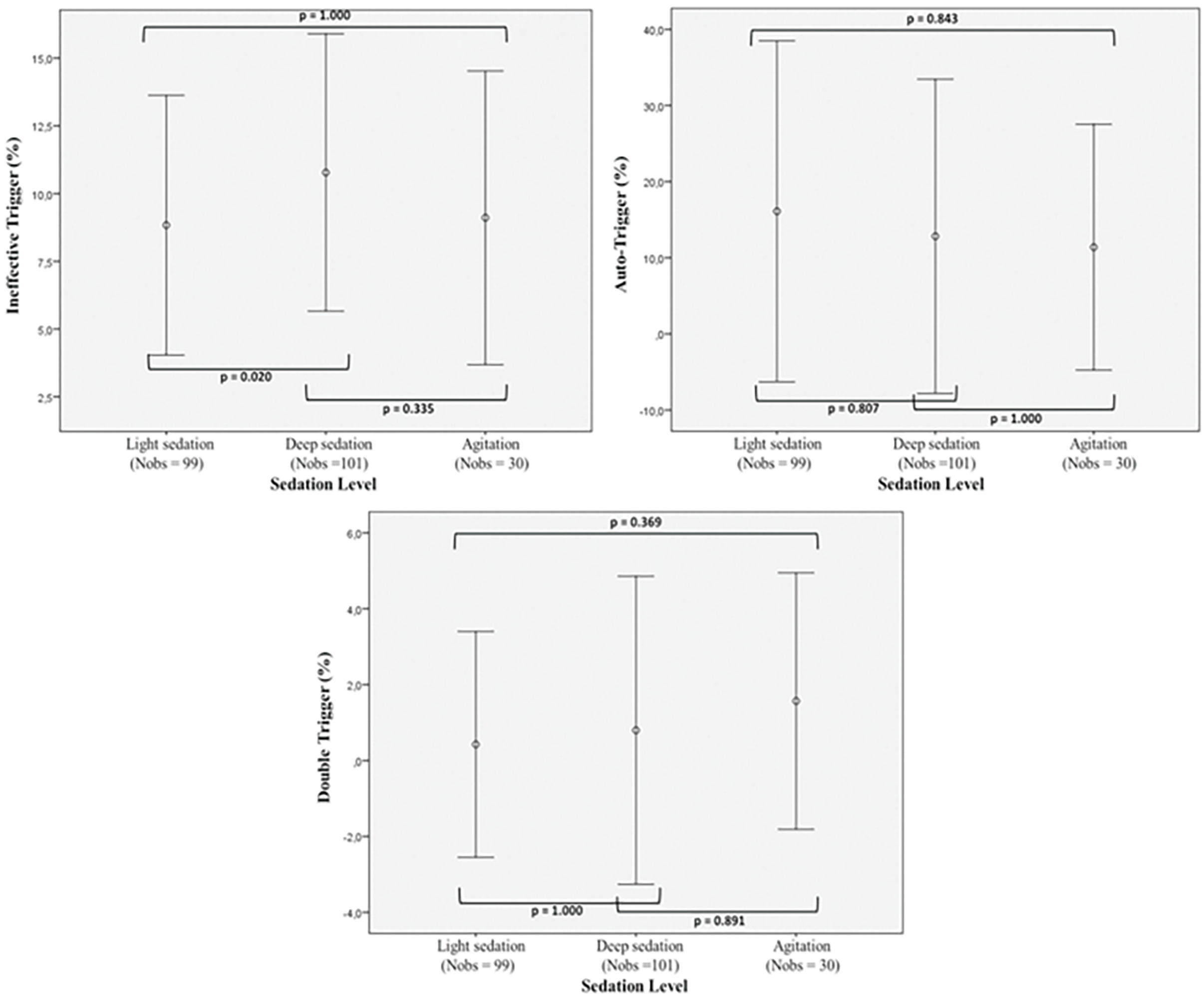

Figure 9S - Bonferroni *post hoc* analysis to evaluate the relationship between the asynchrony index and the level of sedation (patient–ventilator asynchrony due to trigger). N_{obs} -number of observations.

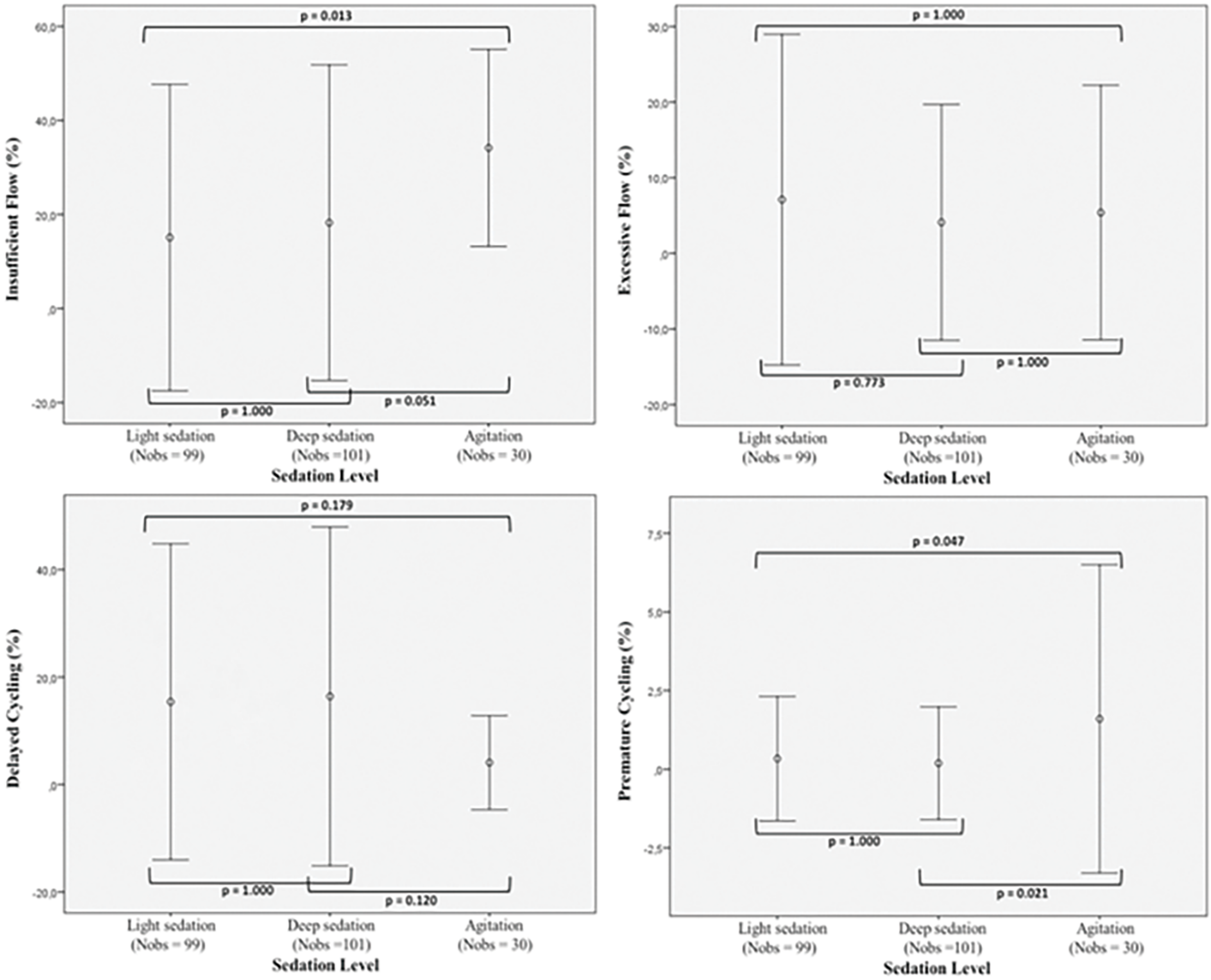

Figure 10S - Bonferroni *post hoc* analysis to evaluate the relationship between the asynchrony index and the level of sedation (patient–ventilator asynchrony due to flow and cycling). N_{obs} - number of observations.

Table 1S - Univariate analysis of the factors related to mortality in the intensive care unit

Variables	Total n = 122	Deceased n = 88	Alive n = 34	p value
Age, years	62.0 ±15.9	64.8 ± 15.2	55.1 ± 15.9	0.003
Male sex	82 (67.2)	57 (64.8)	25 (73.5)	0.358
BMI, kg/m^2	23.2 ± 6.3	23.1 ± 5.8	24.3 ± 6.7	0.329
Type of patient				0.004
Surgical	50 (41.0)	29 (33.0)	21 (61.8)	
Nonsurgical	72 (59.0)	59 (67)	13 (38.2)	
Sepsis	74 (60.7)	65 (73.9)	9 (26.5)	< 0.0001
Pneumonia	24 (19.7)	21 (23.9)	3 (8.8)	0.062
Septic shock	51 (41.8)	45 (51.1)	6 (17.6)	0.001
ARDS	45 (36.9)	39 (44.3)	6 (17.6)	0.006
Sedation	68 (55.7)	55 (62.5)	13 (38.2)	0.016
RASS scale, points	-2.9 ± 2.2	-3.3 ± 2.0	-2.1 ± 2.5	0.014
SOFA, points	5.5 ± 2.8	6.5 ± 2.3	3.2 ± 2.4	< 0.0001
APACHE II, points	19.1 ± 6.5	20.9 ± 6.3	14.5 ± 4.6	< 0.0001

BMI - body mass index; ARDS - acute respiratory distress syndrome; RASS scale - Richmond Sedation and Agitation Scale; SOFA - Sequential Organ Failure Assessment; APACHE - Acute Physiology and Chronic Health Evaluation. The results are expressed as the mean ± standard deviation or n (%).

Table 2S - Multivariate logistic regression analysis (final model) of the factors related to mortality in the intensive care unit

Variables	OR	95% CI	p value
Type of patient	4.20	1.24 - 14.24	0.021
Sepsis	3.94	1.19 - 13.08	0.025
APACHE II	1.16	1.03 - 1.29	0.011
SOFA	1.75	1.30 - 2.36	< 0.0001

OR - odds ratio; CI - confidence interval; APACHE - Acute Physiology and Chronic Health Evaluation; SOFA - Sequential Organ Failure Assessment.

Table 3S - Relationship between sedation level and patient–ventilator asynchrony subtypes

Variables	Light sedation N_{obs} = 99	Deep sedation N_{obs} = 101	Agitation N_{obs} = 30	p value
Ineffective trigger	8.8 ± 4.8	10.8 ± 5.1	9.1 ± 5.4	0.020
Auto-trigger	16.1 ± 22.4	12.8 ± 20.6	11.4 ± 16.1	0.411
Double trigger	0.4 ± 3.0	0.8 ± 4.1	1.6 ± 3.4	0.296
Insufficient flow	15.1 ± 32.6	18.3 ± 33.6	34.2 ± 21.0	0.016
Excessive flow	7.1 ± 21.9	4.1 ± 15.6	5.4 ± 16.9	0.526
Delayed cycling	15.4 ± 29.4	16.4 ± 31.5	4.1 ± 8.8	0.108
Premature cycling	0.3 ± 2.0	0.2 ± 1.8	1.6 ± 4.9	0.023

N_{obs} - number of observations. The results are expressed as the mean ± standard deviation.

Table 4S - Univariate analysis of the relationship between persistent severe patient–ventilator asynchrony and clinical outcomes

Severe patient–ventilator asynchrony on the 1st day of invasive mechanical ventilation and persisting on the 3rd day								
		1st day				3rd day		
Variables	Total n = 122	Severe PVA			Total n = 92	Severe PVA		
		Yes n = 54	No n = 68	p value		Yes n = 46	No n = 46	p value
ΔSOFA, points	-	-	-	-	1.3 ± 1.2	1.6 ± 1.1	1.0 ± 1.2	0.026
Ventilator-associated pneumonia	39 (32.0)	20 (37.0)	16 (23.5)	0.106	28 (30.4)	18 (39.1)	10 (21.7)	0.071
Failed SBT *	20 (16.4)	12 (22.2)	8 (11.8)	0.123	14 (15.2)	11 (23.9)	3 (6.5)	0.021
Ventilation time, days	9.5 ± 9.9	12.2 ± 10.9	7.3 ± 8.4)	0.009	9.0 ± 9.2	11.4 ± 9.6	6.6 ± 8.3	0.013
ICU stay, days	10.6 ± 9.6	12.5 ± 10.8	9.1 ± 8.3)	0.059	11.5 ± 9.6	14.1 ± 9.9	8.8 ± 8.6	0.007
Mortality in the ICU	88 (72.1)	48 (88.9)	40 (58.8)	< 0.0001	68 (73.9)	39 (84.8)	29 (63.0)	0.018
**Persistent severe patient–ventilator asynchrony on the 5th and 7th day of invasive mechanical ventilation**								
		5th day				7th day		
Variables	Total n = 72	Severe PVA			Total n = 50	Severe PVA		
		Yes n = 37	No n = 35	p value		Yes n = 27	No n = 23	p value
ΔSOFA, points	0.9 ± 1.9	1.7 ± 0.9	0.1 ± 2.2)	< 0.0001	1.0 ± 1.8	1.7 ± 0.9	0.2 ± 2.2	0.004
Ventilator-associated pneumonia	22 (30.6)	16 (43.2)	6 (17.1)	0.017	19 (38.0)	14 (51.9)	5 (21.7)	0.030
Failed SBT *	13 (18.1)	10 (27.0)	3 (8.6)	0.043	11 (22.0)	9 (33.3)	2 (8.7)	0.038
Ventilation time, days	12.9 ± 10.2	15.1 ± 9.7	10.6 ± 10.4	0.058	13.1 ± 10.6	15.7 ± 9.4	10.0 ± 11.4	0.060
ICU stay, days	14.9 ± 9.4	17.2 ± 8.9	12.5 ± 9.4	0.034	15.4 ± 9.6	18.2 ± 8.4	12.1 ± 10.1	0.024
Mortality in the ICU	47 (65.3)	32 (86.5)	15 (42.9)	< 0.0001	35 (70.0)	23 (85.2)	12 (52.2)	0.012

PVA - patient–ventilator asynchrony; SOFA - *Sequential Organ Failure Assessment*; SBT - spontaneous breathing test; ICU - intensive care unit. * The spontaneous breathing test was performed with support pressure = 8 cmH_2O and PEEP = 5 cmH_2O in 11 cases and with a "T" tube in nine cases. The results are expressed as the mean ± standard deviation or n (%).
